# The Therapeutic Intervention of Sex Steroid Hormones for Sarcopenia

**DOI:** 10.3389/fmed.2021.739251

**Published:** 2021-10-25

**Authors:** Le-Tian Huang, Jia-He Wang

**Affiliations:** ^1^Department of Oncology, Shengjing Hospital of China Medical University, Shenyang, China; ^2^Department of Family Medicine, Shengjing Hospital of China Medical University, Shenyang, China

**Keywords:** sarcopenia, aging, sex steroid hormones, androgen, estrogen, progesterone, dehydroepiandrosterone, drug

## Abstract

Sarcopenia, characterized by the excessive loss of skeletal muscle mass, strength, and function, is associated with the overall poor muscle performance status of the elderly, and occurs more frequently in those with chronic diseases. The causes of sarcopenia are multifactorial due to the inherent relationship between muscles and molecular mechanisms, such as mitochondrial function, inflammatory pathways, and circulating hormones. Age-related changes in sex steroid hormone concentrations, including testosterone, estrogen, progesterone, and their precursors and derivatives, are an important aspect of the pathogenesis of sarcopenia. In this review, we provide an understanding of the treatment of sarcopenia through the regulation of sex steroid hormones. The potential benefits and future research emphasis of each sex steroid hormone therapeutic intervention (testosterone, SARMs, estrogen, SERMs, DHEA, and progesterone) for sarcopenia are discussed. Enhanced understanding of the role of sex steroid hormones in the treatment for sarcopenia could lead to the development of hormone therapeutic approaches in combination with specific exercise and nutrition regimens.

## Introduction

The term “sarcopenia,” established based on the efforts of different groups, is typically defined as an age-related decline in skeletal muscle mass and muscle function (1–4). It is characterized by the progressive loss of muscle mass, strength, endurance, and reduced metabolic capacity of muscle fibers (5). The average sarcopenia incidence is 5–13% for people aged 60–70 years and 11–50% for those older than 80 years (6). It is associated with many age-related adverse events, such as an increase in the duration of hospitalization (7, 8), an impaired quality of life (9), reduced mobility (8), and a higher risk of hip or vertebral fractures (10, 11). In some instances, sarcopenia is secondary to some uncontrolled chronic diseases, such as malignant tumors, liver disease, or renal dysfunction. Moreover, progressive sarcopenia may worsen the primary disease, or even increase the rate of mortality (12, 13). Currently, the common ways to treat sarcopenia have been approved as follows: aerobic and resistance exercise, protein and energy intake, and vitamin D supplementation, but with limited therapeutic effects (14, 15). Over time, sarcopenia has become a vital public health issue and is now recognized as an independently reportable clinical disorder, and better treatments are urgently needed (16).

The pathophysiology of sarcopenia is both complex and multidirectional. Reduced muscle mass in the elderly can be ascribed to the lower rate of muscle protein synthesis when compared to the rate of muscle proteolysis (17). Microscopically, atrophy of type II muscle fibers increases, and motor units within the muscle are lost and replaced by adipose and connective tissue (18). As a result, abnormal muscle metabolism, reduced muscle mass, and muscle strength capacity as well as increased muscle damage is observed (18).

The severity of sarcopenia can be measured using three parameters: muscle strength, muscle mass, and physical performance. Hand grip strength, leg muscle strength, and a chair stand test are applied to test muscle strength (19). Low muscle mass is estimated by the lumbar muscle cross-sectional area derived from computed tomography (CT) or magnetic resonance imaging (MRI) (20), or is assessed by clinical practice, such as the appendicular skeletal muscle mass (ASMM) (21, 22). Lean body mass (LBM) is often used to measure muscle mass, especially in the condition of sarcopenia obesity (1). Physical performance is a multidirectional concept involving muscle function and nervous system function, including the body balance (23). It can be variously measured by the timed-up and go test (TUG), the 6-min walk test, and the short physical performance battery (SPPB) (1).

Research exploring the inner mechanisms of sarcopenia is expanding and includes studies on muscle fiber composition and neuromuscular function, the potential of myo-satellite cell to proliferate and differentiate, inflammatory processes, mitochondrial dysfunction, as well as the age-related alterations of anabolic hormones in the endocrine system (24–27). Muscle mass is associated with a dynamic change in the anabolic and catabolic processes of skeletal muscle tissues (27). Anabolic hormones, such as sex steroid hormones, growth hormone, and insulin, are believed to play a major role in muscle tissue growth and remodeling (27).

**Figure 1:**
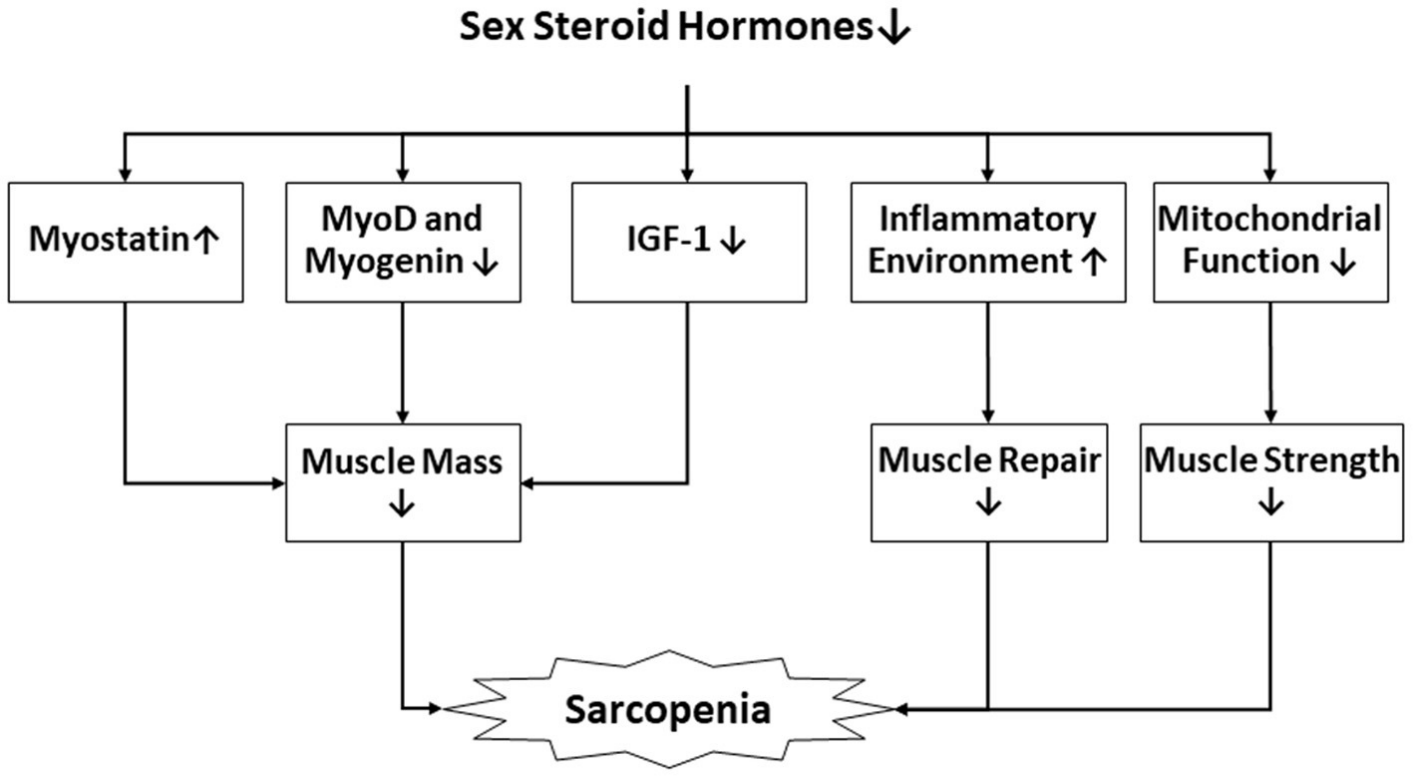
The interactions between sex steroid hormones and sarcopenia. IGF-1, Insulin-like growth factor-1.

Sex steroid hormones such as testosterone, estrogen, and progesterone, are important systemic anabolic hormones involved in maintenance of skeletal muscle mass and function, including hypertrophy and the regeneration of damaged muscles (28, 29). During aging, their levels become depleted, which is consistent with decreased muscle mass (29). Myo-satellite cells, myoblasts and myocytes both express androgen receptors and estrogen receptors, while progesterone receptors are found in myocytes. It has been found that the expression of the receptors in muscle cells plays an essential role in regulating the proliferation and differentiation of these cells (30–32). Moreover, these sex steroid hormones may participate in intracellular signaling pathways such as the IGF-1/Akt/mTOR pathway, MAPK pathway, Wnt and Notch signaling, among others, either positively or negatively, although the specific mechanism is still not well-understood (33–36). In addition, suppression of myostatin mRNA expression, activation of MyoD and myogenin, improved function of mitochondrial, and counteracting inflammation play roles in the physiological effects of sex steroid hormones in the skeletal muscle cells (Table 1, Figure 1) (29, 34, 37–39).

**Table 1:** Physiological effects of sex steroid hormones on receptors expression, signaling pathways, and metabolic alterations in the skeletal muscles.

	**Receptors expression in muscle cells**	**IGF-1/Akt/mTOR pathway**	**MAPK pathway**	**Wnt and notch pathway**	**Myostatin**	**MyoD and myogenin**	**IGF-1**	**Inflammatory environment**	**Mitochondrial function**
Testosetone	+	+	+	+	+	ND	+	+	+
Estrogen	+	+	+	ND	+	+	+	+	+
Progesterone	+	ND	ND	ND	ND	+	ND	ND	+
DHEA	ND	ND	ND	ND	ND	ND	+	ND	+

*DHEA, Dehydroepiandrosterone; IGF-1, Insulin-like growth factor-1; ND, No available data*.

Decreased circulating levels of the sex steroids may lead to deterioration in muscle mass and function in elderly (40). Although sex hormone-derived drugs have not been widely used, it has been demonstrated that supplemental hormone intervention could be an effective approach to treat sarcopenia (41). In this review, we will outline the role of sex steroid hormones in the treatment of sarcopenia, focusing on recent findings.

## Testosterone

Testosterone, a representative sex steroid hormone, is mainly produced by male Leydig cells and female ovarian thecal cells, and partly by adrenal gland (42). As the principal physiological anabolic hormone, testosterone increases protein synthesis in skeletal muscle, promotes muscle regeneration and repair by activation of myo-satellite cells, counteracts muscle proteolysis, and increases intramuscular insulin-like growth factor-1 (IGF-1) levels, etc. (43–45). During aging, testosterone levels in healthy men fall by 1% annually from the age of 30. For women, the testosterone levels at 40 years of age are about 50% of those at 20 years of age (46, 47). In some clinical conditions, the reductions in the testosterone levels may result in poorer clinical prognosis (48, 49).

Considering the significant physiological functions of testosterone, and its consistent benefits on muscle mass and strength in hypogonadal treatment, multiple randomized controlled trials (RCTs) have been conducted to explore the effects of testosterone replacement therapy (TRT) on the elderly since the 1990s. However, the effects observed on muscle performance and physical function were inconsistent due to the different treatment methods employed (47, 48, 50, 51). For healthy older men, TRT can help increase LBM, but muscle strength does not change (50, 52). Some other clinical trials showed that testosterone substitution results in an increase of both LBM and muscle strength (53–55). Previous trials were also limited by their relatively short duration, small sample sizes, and the heterogeneity of testosterone doses, regimens, and on-treatment testosterone levels (50–54). A meta-analysis conducted in 2018 showed that TRT may increase physical performance (measured by the Physical Activity Scale for the Elderly score and the 6-min walk test), but failed to increase muscle strength (measured by leg muscle strength and hand grip strength) (56).

The positive effects of exercise and physical training, including slowing down muscle loss and improving strength and performance in the elderly, have been substantiated (57, 58). Physical training, as the most effective intervention for sarcopenia, is often used in combination with pharmacological interventions to improve the effectiveness. Although, previous studies of TRT combined with exercise presented inconsistent results (55, 56, 59, 60), several clinical trials showed that the combination treatment can help increase the muscle mass or strength (59, 60). A prospective study demonstrated that TRT offers no benefit beyond resistance exercise alone (61). In 2021, a systematic review and meta-analysis on TRT of 21 RCTs for LBM and 15 RCTs for muscle strength concluded that both LBM and muscular strength were significantly improved by a combination regimen, which included physical exercise and testosterone-based interventions, when compared with physical exercise or testosterone-based interventions alone (62).

Several studies showed that patients with chronic diseases or injury can benefit from TRT (63, 64). Resistance training combined with TRT maximized improvements in muscle mass when compared with TRT alone for patients with spinal cord injury (63). In patients with advanced cancer, TRT improved LBM and physical activity, as well as their quality of life compared to placebo (64).

Recent studies in elderly women explored the anabolic effect of testosterone on sarcopenia (65, 66). Compared with placebo, low doses of TRT in women elevated the serum testosterone concentration and induced a significant increase in total LBM, and showed microscopic type II muscle fiber hypertrophy induced by testosterone (65). Another study showed that short-term TRT in hysterectomized women with low circulating testosterone levels was related to increased trunk muscle area (66).

Nevertheless, TRT is still not widely recommended due to the adverse effects. TRT is associated with a higher risk of urogenital problems including benign prostatic hyperplasia and prostate cancer (67). Additionally, adverse cardiovascular events such as a significant increase in coronary artery non-calcified plaque volume (68) and menstrual changes in women and gynecomastia in men (69) are known to occur. A recent meta-analysis of testosterone did not indicate significant side effects and suggested that the adverse effects may be decreased by avoiding supraphysiological levels of plasma testosterone (62).

The strong anabolic effects of TRT, especially when combined with physical exercise, on muscle mass have been confirmed by a large body of evidence and the effects on muscle strength and function are supported by recent studies. Due to its inevitable side effects, TRT is still not recommended for wide use. Short term or intermittent TRT to maintain low serum testosterone levels in circulation may help lower incidence of adverse events. In the future, the risk-benefit balance between the adverse effects and effectiveness of TRT for sarcopenia needs to be explored. Furthermore, a specific TRT regimen (dosage for hormone therapy, in combination with exercise or nutrition) for older sarcopenic patients with different health conditions is worth exploring.

## Selective Androgen Receptor Modulators (SARMs)

As an alternative to androgen replacement therapy, SARMs were created to provide a targeted therapeutic effect through the androgen receptors in different tissues. Binding of SARMS to androgen receptor in the prostate and seminal vesicles is partial agonistic, while in muscle and bone it is fully agonistic (70). Some SARMs have an anabolic/androgenic ratio of 20:1, in contrast to the ratio of 1:1 for testosterone (71).

The effects of highly selective drugs have been analyzed in several preclinical studies (72–75). Among them, one study demonstrated increased gastrocnemius muscle weight, bone biomechanical properties, and bone mineral density in ovariectomized female rats that were administered SARMs (72). On this basis, several clinical trials have found SARMs to benefit with body composition, exhibiting increased muscle mass, and decreased fat quality. However, its effects on muscle strength and function and its long-term efficacy remain inconclusive (70, 76–79). In a phase II clinical trial, Dalton JT showed that enobosarm (SARM) resulted in dose-dependent improvements in both LBM and physical performance as evaluated by the stair climbing test in healthy postmenopausal women and in aged men (80). Nevertheless, enobosarm failed to improve stair climb power in a separate phase III clinical trial, although LBM was increased significantly (81, 82).

According to various clinical trials, postmenopausal women could also take advantage of SARMs treatment. Neil D suggested that healthy postmenopausal women presented higher sensitivity to LBM improvement when treated with GSK2881078 (SARM) than healthy elderly men. Furthermore, the female cohort presented a better response at lower doses than males (73). In addition to postmenopausal women, patients suffering from any chronic disease, such as osteoporosis, non-small-cell lung cancer, stress urinary incontinence, and hip fractures, can also benefit from SARMs treatment with increased muscle mass (70, 81). Besides, the side effects of SARMs are much milder than TRT, such as the increases in alanine transaminase and aspartate transaminase, redeuctions in high density lipoprotein, and suppression of sex hormone binding globulin and total testosterone levels (76–78, 80).

Although SARMs have been shown to increase muscle mass, food and drug administration (FDA) still does not approve SARMs for the treatment of sarcopenia despite the fewer side effects when compared with testosterone. This may be a result of the inconsistent results of SARMs on muscle function. However, given the potential and safety of SARM therapy, future RCTs should be continued. It is recommended that SARMs be combined with nutritional supplements or exercise training, which will contribute to increased LBM and muscle function, especially for those with chronic diseases.

## Estrogen

Estrogen, a common sex steroid hormone active in the reproductive organs, is also found in other organs and tissues, such as the skeletal muscle (83). In postmenopausal women or following bilateral oophorectomy, limited estrogen levels can cause osteoporosis, frailty, and sarcopenia (84–86). Estrogen deficiency is also related to decreased muscle strength (87), but the effects and function of estrogen on muscles remain unclear (88, 89). Estrogen may reduce the inflammation in the surrounding environment and thus prevent depletion of satellite cells, which help enhance skeletal muscle repair and growth if provided with necessary precursors (90).

Mice with low estrogen levels showed a decline in skeletal muscle strength, and this effect could be reversed by estrogen replacement therapy (ERT) (91, 92). Nevertheless, results concerning ERT in humans are conflicting (42). Although postmenopausal estrogen therapy is not as potent as testosterone, some studies revealed that it had a positive effect on muscle strength or muscle function (42, 93–97). However, other studies didn't support the hypothesis that ERT could work against sarcopenia (98–100). A meta-analysis of 24 studies showed that postmenopausal women treated with ERT had a significant impact on muscle strength compared with placebo (94). A subsequent meta-analysis in 2019, which included 12 studies, looked at the effects of hormones on muscle mass. No significant differences between an estrogen-based or an estrogen-progesterone-based hormone therapy and placebo were observed (101). Although the type and dosage of estrogen replacement administered varied among different trials, there were still no definitive conclusions.

Similar to TRT, ERT has been shown to enhance muscle function when combined with resistance training (94). In a randomized placebo-controlled study in postmenopausal women, estrogen-progesterone-based hormone therapy combined with high-impact physical exercise showed significant increases in muscle strength as evaluated by knee extension torque and vertical jumping height when compared with the control group (99); more research is necessary to assess these findings.

Some severe complications have been reported when ERT is used in postmenopausal women with sarcopenia, including a higher risk of cardiovascular disease, breast cancer, endometrial cancer, and deep vein thrombosis (42, 102, 103). In a large-scale clinical trial (16,608 postmenopausal women aged 50–79 years), estrogen plus progestin increased total and invasive breast cancers compared with placebo during 5 years (104). Since ERT does not translate into improved physical function, and has potential risks, ERT is not recommended for healthy postmenopausal women to prevent or treat sarcopenia. Future clinical trials of estrogen as a therapeutic intervention for sarcopenia need to be conducted cautiously.

## Selective Estrogen Receptor Modulators (SERMs)

SERMs are similar to estrogen in the observed drug effects but with fewer side effects. The highly selective drugs have estrogenic effects on the bone composition, blood vessels, and lipid metabolism but show anti-estrogenic effects on the breast and genital system (105, 106), leading to fewer side effects compared to estrogen. In a preclinical study, estradiol and SERMs influenced ER-α function in hSkM cells to promote muscle growth in postmenopausal women (107). Studies in ovariectomized rats have shown that raloxifene (SERM) modifies body composition by reducing body fat accumulation (108).

A RCT demonstrated that a 1-year raloxifene treatment could significantly increase the fat-free mass compared to placebo for postmenopausal women, without providing any significant advantage in muscle strength (109). In another randomized placebo-controlled trial, 1-year raloxifene treatment prevented enhanced body weight and abdominal adiposity (110). A study in 2016 consisting of 4,383 patients suggested that 5-year raloxifene treatment had significant effects on body composition achieved through maintaining body weight and increasing body mass index, with only minor side effects (111). Although it did not directly assess the roles of SERM on muscle mass or muscle strength, the 5-year trial revealed a preferential effect of SERM on the maintenance of muscle mass with fewer adverse effects. The side effects of SERMs were not significant compared with those of control groups, notably leg cramps and hot flashes (111).

Although there is still insufficient evidence on the effect of SERM on muscle mass and strength, it has been shown to maintain weight and increase fat-free mass over a long period of time in older postmenopausal women, with minor side effects. For this group, further research to explore the long-term effects of SERM on muscle mass and/or mass function may be useful, with consideration given to combining SERM with exercises, or for patients with chronic diseases who overtime, lose weight.

## Dehydroepiandrosterone (DHEA)

DHEA is mainly produced by the adrenal glands and can be converted to active androgens or estrogens in other tissues (112, 113). As a natural precursor to steroid hormone, DHEA exerts its anabolic effects when metabolized to active androgens or estrogens. DHEA also stimulates the production of IGF-1, which helps in muscle growth and repair, and increases its bioavailability in muscles by lowering the levels of IGF-1 binding protein-1 (114–116). In addition, DHEA improved insulin sensitivity, which directly affects the anabolic efficacy, by increasing the absorption rate of amino acids in the skeletal muscle (117). DHEA levels decline with age (118), and men in their 70–80s only have ~20% of their peak value of DHEA, while similarly aged women have 30% of their peak value of DHEA (119). The decline in the level of DHEA is related to the loss of age-related muscle mass and strength (112, 113, 120, 121).

Thus far, the efficacy of DHEA replacement therapy for sarcopenia has not been consistently demonstrated. A randomized placebo-controlled trial showed that a daily oral dose of 100 mg was effective in maintaining body fat and muscle strength in elderly men, but not in elderly women (122). A decrease in body fat was observed in some studies (123, 124), while, other studies showed slight positive effects on muscle mass, strength, or function (125, 126). In 2011, a systematic review involving eight studies failed to confirm any efficacy of DHEA administration on muscle strength or physical performance (127). A meta-analysis in 2018, comprising four RCTs showed no positive effects of DHEA on muscles (128).

It is worth noting that some research found that when combined with exercise training, DHEA may play a better role in increasing muscle mass and strength in both rats and humans (129, 130). Elderly people in a RCT benefitted from a regimen with 50 mg DHEA daily and weightlifting exercise. This study showed a significant increase in muscular strength and mass in the combination group compared with individuals in the non-combination group (130). However, such a synergistic effect of DHEA was not confirmed by another combination trial (126). The differences between the results may be associated with the diversity of research designs, particularly the treatment time and dose, and the size of the sample population.

DHEA, which has fewer side effects than testosterone, is generally well-tolerated. Minor side effects including edema, facial hair growth, acne, and seborrhea have been observed in both men and women (131, 132). Unlike testosterone, no significant prostatic adverse events of DHEA were observed in elderly men. Most of these studies revealed no positive effects from DHEA supplementation alone. This was supported by a recent high quality meta-analysis (128). When DHEA was combined with exercises, muscle mass, and strength of elderly people was enhanced in some instances. As DHEA was observed to decrease the body fat in several trials, future RCT studies should explore an optimal formulation combining an exercise regimen with DHEA supplementation for senior patients with high body weight.

## Progesterone

Progesterone, commonly produced by the adrenal gland as well as the ovaries or testes, has important functions in the female reproductive system and mammary gland. It also has functions involving several other tissues, including the cardiovascular system, the central nervous system, bones, and muscles (133). Progesterone receptors and estrogen receptors which are located and expressed in skeletal muscle tissue exert their direct effects on muscle tissue (134–136).

Progestogens have not been approved for use for sarcopenia, because of insufficient research on their direct therapeutic effect on muscle mass or function. In postmenopausal women, similar to testosterone, progesterone administration improved muscle protein fractional synthesis rate by ~50% (33). The study also showed that progesterone has potent stimulatory impacts on myogenic differentiation 1 (MYOD1) mRNA expression, which is involved in muscle protein synthesis (33).

Megestrol is a synthetic progestin, which improves appetites and weight gain in patients with cancer, immunodeficiency syndrome, or other disabilities (137, 138). However, a RCT focusing on the elderly who do not have severe chronic illnesses, cancer, or immunodeficiency, showed negative results. The addition of megestrol did not seem to enhance the beneficial effects of resistance muscle training, leading to less muscle strength and function gains (139).

When combined with training and estrogen therapy, synthetic progesterone exerted a synergistic effect in increasing leg muscle cross-sectional area compared with the non-combination therapy group (140). Oral contraceptives (OC), which consists of synthetic estradiol and synthetic progesterone, are widely used by young females. A 2-year RCT indicated that young female runners receiving OC and resistance training increased LBM significantly compared to non-users (141). In contrast, another clinical study showed that OC impaired LBM gains in young women after resistant training, and this was connected to lower levels of anabolic hormones, such as DHEA and IGF-1, but higher catabolic hormone, like cortisol (142).

Adverse events including venous thromboembolism and breast cancer were observed during synthetic progesterone treatment. Compared with synthetic progestin, bioidentical progesterone is safer and more efficient. It has been shown that bioidentical progesterone can lower risk of breast cancer from estrogen (143), however, few studies have explored the effects of bioidentical progesterone on muscles.

The roles of progesterone on muscle mass or muscle function are controversial, even when combined with exercises. Based on this, we do not recommend progesterone supplementation for patients with muscle loss. Future research could focus on the use of bioidentical progesterone, especially in elderly patients with a poor appetite or low body weight.

## Outlook

Both positive and negative effects are observed in treatments using sex steroid supplementation for sarcopenia, namely testosterone, SARMs, estrogen, SERMs, DHEA, and progesterone. The main effects and our suggestions are shown in Table 2. It can be inferred that elderly with sarcopenia with different health conditions may benefit from different hormone treatments and future trials are needed to explore.

**Table 2:** Summary of the main effects of sex steroid hormones.

	**Muscle mass**	**Muscle strength**	**Physical performance**	**Adverse events**	**Effects when combined with exercises**	**Patients who may benefit**	**Future directions**
Testosterone	Sufficient evidence in favor	Some evidence in favor	Some evidence in favor	Gynecomastia and prostatic diseases in men; menstrual changes in women; cardiovascular events	Sufficient evidence in favor	Hypogonadal men; short term or intermittent TRT for elderly men and postmenopausal women, including patients with chronic diseases or injury	Specific TRT formulation (dosage for hormone therapy, in combination with exercise or nutrition) for older sarcopenic people with different health conditions
SARMs	Sufficient evidence in favor	Insufficient evidence	Insufficient evidence	Increase in alanine transaminase and aspartate transaminase	No evidence	Elderly men and postmenopausal women, especially with chronic diseases	The long-term effects of SARMs combined with exercise training on muscle mass and/or mass function of elderly with chronic diseases
Estrogen	Insufficient evidence	Insufficient evidence	Insufficient evidence	Breast cancer, endometrial cancer, cardiovascular events, deep vein thrombosis	Some evidence in favor	Not recommended	Need to be conducted cautiously
SERMs	Some evidence in favor	Insufficient evidence	Insufficient evidence	Leg cramps, hot flashes	No evidence	Postmenopausal women who need long time administration to maintain their body weight and muscle mass	The long-term effects of SERMs on muscle mass and/or mass function of elderly with chronic diseases who are under normal weight over time
DHEA	Insufficient evidence	Insufficient evidence	Insufficient evidence	Edema, facial hair growth, acne, and seborrhea	Insufficient evidence	Not recommended	The optimal formulation of exercise and DHEA for specific high body weight seniors
Progesterone	Insufficient evidence	Insufficient evidence	Insufficient evidence	Venous thromboembolism, breast cancer	Insufficient evidence	Not recommended	The effects of bioidentical progesterone on postmenopausal women, especially with poor appetite or low body weight

*DHEA, Dehydroepiandrosterone; SARMs, Selective androgen receptor modulators; SERM, Selective estrogen receptor modulators; TRT, testosterone replacement therapy*.

## Conclusion

The decline of sex steroid hormones during aging influences the maintenance and development of skeletal muscles, resulting in sarcopenia. However, no sex steroid supplementation, including testosterone, estrogen, and progesterone, has been approved by the FDA for the management of sarcopenia, due to insufficient current evidence or low safety and effectiveness. In recent years, studies have explored better formulations of sex steroid hormones for sarcopenia, revealing more possibilities to treat this age-related disease. TRT is a promising treatment for sarcopenia, and we suggest that short term or intermittent TRT to maintain low serum testosterone levels may assist in optimizing its safety and effectiveness. Therefore, a specific TRT formulation for older sarcopenic patients with different health conditions is worth exploring. It is recommended that SARMs be combined with exercise training, especially for those with chronic diseases. Given the potential and safety of SARM therapy, future RCTs should be continued. SERMs can be used to maintain weight and increase fat-free mass over a long period of time in postmenopausal women with minor side effects. Estrogen is not recommended to treat sarcopenia or for further exploration due to its severe adverse effects. The role of progesterone and DHEA in the treatment of sarcopenia is still unclear. Large-scale clinical studies are necessary to investigate the promising drugs: testosterone, SARM and SERM. Considering the individual patient characteristics (sex, age, and chronic diseases), trials of different specific sex steroid interventions (combined with physical exercises or nutrition) for management of sarcopenia are urgently needed to determine their effectiveness and safety. Enhanced understanding of the role of sex steroid hormones in the treatment for sarcopenia could lead to the development of hormone therapeutic approaches, which could benefit patients with sarcopenia.

## Author Contributions

All authors contributed to the article and approved the submitted version.

## Funding

This work was supported by Key Research and Development Program of Liaoning Province (2019JH8/10300021).

## Conflict of Interest

The authors declare that the research was conducted in the absence of any commercial or financial relationships that could be construed as a potential conflict of interest.

## Publisher's Note

All claims expressed in this article are solely those of the authors and do not necessarily represent those of their affiliated organizations, or those of the publisher, the editors and the reviewers. Any product that may be evaluated in this article, or claim that may be made by its manufacturer, is not guaranteed or endorsed by the publisher.

## References

[1] Cruz-Jentoft AJ Bahat G Bauer J Boirie Y Bruyère O Cederholm T. Sarcopenia: revised European consensus on definition and diagnosis. Age Ageing. (2019) 48:16–31. 10.1093/ageing/afz046 30312372 PMC6322506

[2] Chen LK Woo J Assantachai P Auyeung TW Chou MY Iijima K. Asian Working Group for Sarcopenia: 2019 consensus update on sarcopenia diagnosis and treatment. J Am Med Dir Assoc. (2020) 21:300–7.e2. 10.1016/j.jamda.2019.12.012 32033882

[3] McLean RR Shardell MD Alley DE Cawthon PM Fragala MS Harris TB. Criteria for clinically relevant weakness and low lean mass and their longitudinal association with incident mobility impairment and mortality: the foundation for the National Institutes of Health (FNIH) sarcopenia project. J Gerontol A Biol Sci Med Sci. (2014) 69:576–83. 10.1093/gerona/glu012 24737560 PMC3991140

[4] Studenski SA Peters KW Alley DE Cawthon PM McLean RR Harris TB. The FNIH sarcopenia project: rationale, study description, conference recommendations, and final estimates. J Gerontol A Biol Sci Med Sci. (2014) 69:547–58. 10.1093/gerona/glu010 24737557 PMC3991146

[5] Cruz-Jentoft AJ Bahat G Bauer J Boirie Y Bruyère O Cederholm T. Sarcopenia: revised European consensus on definition and diagnosis. Age Ageing. (2019) 48:16–31. 10.1093/ageing/afy169 30312372 PMC6322506

[6] Papadopoulou SK. Sarcopenia: a contemporary health problem among older adult populations. Nutrients. (2020) 12:1293. 10.3390/nu12051293 32370051 PMC7282252

[7] Ligthart-Melis GC Luiking YC Kakourou A Cederholm T Maier AB. de van der Schueren MAE. Frailty, sarcopenia, and malnutrition frequently (co-)occur in hospitalized older adults: a systematic review and meta-analysis. J Am Med Dir Assoc. (2020) 21:1216–28. 10.1016/j.jamda.2020.03.006 32327302

[8] Chung GE Park HE Lee H Kim MJ Choi S-Y Yim JY. Sarcopenic obesity is significantly associated with coronary artery calcification. Front Med. (2021) 8:651961. 10.3389/fmed.2021.651961 33855037 PMC8039284

[9] Argilés JM Busquets S Stemmler B López-Soriano FJ. Cancer cachexia: understanding the molecular basis. Nat Rev Cancer. (2014) 14:754–62. 10.1038/nrc3829 25291291

[10] Hida T Shimokata H Sakai Y Ito S Matsui Y Takemura M. Sarcopenia and sarcopenic leg as potential risk factors for acute osteoporotic vertebral fracture among older women. Eur Spine J. (2016) 25:3424–31. 10.1007/s00586-015-3805-5 25690348

[11] Chiles Shaffer N Huang Y Abraham DS Cheng YJ Lu W Gruber-Baldini AL. Comparing longitudinal sarcopenia trends by definitions across men and women after hip fracture. J Am Geriatr Soc. (2020) 68:1537–44. 10.1111/jgs.16417 32239496 PMC7416476

[12] Nishikawa H Shiraki M Hiramatsu A Moriya K Hino K Nishiguchi S. Japan Society of Hepatology guidelines for sarcopenia in liver disease (1st edition): recommendation from the working group for creation of sarcopenia assessment criteria. Hepatol Res. (2016) 46:951–63. 10.1111/hepr.12774 27481650

[13] Welch C. K Hassan-Smith Z, A Greig C, M Lord J, A Jackson T. Acute sarcopenia secondary to hospitalisation - an emerging condition affecting older adults. Aging Dis. (2018) 9:151–64. 10.14336/AD.2017.0315 29392090 PMC5772853

[14] Dhillon RJ Hasni S. Pathogenesis and management of sarcopenia. Clin Geriatr Med. (2017) 33:17–26. 10.1016/j.cger.2016.08.002 27886695 PMC5127276

[15] Sgrò P Sansone M Sansone A Sabatini S Borrione P Romanelli F. Physical exercise, nutrition and hormones: three pillars to fight sarcopenia. Aging Male. (2019) 22:75–88. 10.1080/13685538.2018.1439004 29451419

[16] Sakuma K Aoi W Yamaguchi A. Molecular mechanism of sarcopenia and cachexia: recent research advances. Pflugers Arch. (2017) 469:573–91. 10.1007/s00424-016-1933-3 28101649

[17] Balagopal P Rooyackers OE Adey DB Ades PA Nair KS. Effects of aging on *in vivo* synthesis of skeletal muscle myosin heavy-chain and sarcoplasmic protein in humans. Am J Physiol. (1997) 273:E790–800. 10.1152/ajpendo.1997.273.4.E790 9357810

[18] Keller K. Sarcopenia. Sarkopenie Wiener medizinische Wochenschrift. (2019) 169:157–72. 10.1007/s10354-018-0618-2 29411194

[19] Merchant RA Chan YH Hui RJY Lim JY Kwek SC Seetharaman SK. Possible sarcopenia and impact of dual-task exercise on gait speed, handgrip strength, falls, and perceived health. Front Med. (2021) 8:660463. 10.3389/fmed.2021.660463 33937294 PMC8086796

[20] Derstine BA Holcombe SA Ross BE Wang NC Su GL Wang SC. Skeletal muscle cutoff values for sarcopenia diagnosis using T10 to L5 measurements in a healthy US population. Sci Rep. (2018) 8:11369. 10.1038/s41598-018-29825-5 30054580 PMC6063941

[21] Schweitzer L Geisler C Pourhassan M Braun W Glüer CC Bosy-Westphal A. What is the best reference site for a single MRI slice to assess whole-body skeletal muscle and adipose tissue volumes in healthy adults? Am J Clin Nutr. (2015) 102:58–65. 10.3945/ajcn.115.111203 26016860

[22] Shen W Punyanitya M Wang Z Gallagher D St-Onge MP Albu J. Total body skeletal muscle and adipose tissue volumes: estimation from a single abdominal cross-sectional image. J Appl Physiol. (2004) 97:2333–8. 10.1152/japplphysiol.00744.2004 15310748

[23] Beaudart C Rolland Y Cruz-Jentoft AJ Bauer JM Sieber C Cooper C. Assessment of muscle function and physical performance in daily clinical practice: a position paper endorsed by the european society for clinical and economic aspects of osteoporosis, osteoarthritis and musculoskeletal diseases (ESCEO). Calcif Tissue Int. (2019) 105:1–14. 10.1007/s00223-019-00545-w 30972475

[24] Marty E Liu Y Samuel A Or O Lane J. A review of sarcopenia: Enhancing awareness of an increasingly prevalent disease. Bone. (2017) 105:276–86. 10.1016/j.bone.2017.09.008 28931495

[25] He WA Berardi E Cardillo VM Acharyya S Aulino P Thomas-Ahner J. NF-κB-mediated Pax7 dysregulation in the muscle microenvironment promotes cancer cachexia. J Clin Invest. (2013) 123:4821–35. 10.1172/JCI68523 24084740 PMC3809785

[26] Wåhlin-Larsson B Carnac G Kadi F. The influence of systemic inflammation on skeletal muscle in physically active elderly women. Age. (2014) 36:9718. 10.1007/s11357-014-9718-0 25311555 PMC4199340

[27] Priego T Martín AI González-Hedström D Granado M López-Calderón A. Role of hormones in sarcopenia. Vitam Horm. (2021) 115:535–70. 10.1016/bs.vh.2020.12.021 33706961

[28] Lecker SH Jagoe RT Gilbert A Gomes M Baracos V Bailey J. Multiple types of skeletal muscle atrophy involve a common program of changes in gene expression. FASEB J. (2004) 18:39–51. 10.1096/fj.03-0610com 14718385

[29] Kim YJ Tamadon A Park HT Kim H Ku SY. The role of sex steroid hormones in the pathophysiology and treatment of sarcopenia. Osteoporos Sarcopenia. (2016) 2:140–55. 10.1016/j.afos.2016.06.002 30775480 PMC6372754

[30] Wannenes F Caprio M Gatta L Fabbri A Bonini S Moretti C. Androgen receptor expression during C2C12 skeletal muscle cell line differentiation. Mol Cell Endocrinol. (2008) 292:11–9. 10.1016/j.mce.2008.05.018 18588941

[31] Sinha-Hikim I Taylor WE Gonzalez-Cadavid NF Zheng W Bhasin S. Androgen receptor in human skeletal muscle and cultured muscle satellite cells: up-regulation by androgen treatment. J Clin Endocrinol Metab. (2004) 89:5245–55. 10.1210/jc.2004-0084 15472231

[32] Kalbe C Mau M Wollenhaupt K Rehfeldt C. Evidence for estrogen receptor alpha and beta expression in skeletal muscle of pigs. Histochem Cell Biol. (2007) 127:95–107. 10.1007/s00418-006-0224-z 16897031

[33] White JP Gao S Puppa MJ Sato S Welle SL Carson JA. Testosterone regulation of Akt/mTORC1/FoxO3a signaling in skeletal muscle. Mol Cell Endocrinol. (2013) 365:174–86. 10.1016/j.mce.2012.10.019 23116773 PMC3529800

[34] Haren MT Siddiqui AM Armbrecht HJ Kevorkian RT Kim MJ Haas MJ. Testosterone modulates gene expression pathways regulating nutrient accumulation, glucose metabolism and protein turnover in mouse skeletal muscle. Int J Androl. (2011) 34:55–68. 10.1111/j.1365-2605.2010.01061.x 20403060

[35] Smith GI Yoshino J Reeds DN Bradley D Burrows RE Heisey HD. Testosterone and progesterone, but not estradiol, stimulate muscle protein synthesis in postmenopausal women. J Clin Endocrinol Metab. (2014) 99:256–65. 10.1210/jc.2013-2835 24203065 PMC3879672

[36] Liu XH Wu Y Yao S Levine AC Kirschenbaum A Collier L. Androgens up-regulate transcription of the Notch inhibitor Numb in C2C12 myoblasts via Wnt/β-catenin signaling to T cell factor elements in the Numb promoter. J Biol Chem. (2013) 288:17990–8. 10.1074/jbc.M113.478487 23649620 PMC3689944

[37] Mendler L Baka Z Kovács-Simon A Dux L. Androgens negatively regulate myostatin expression in an androgen-dependent skeletal muscle. Biochem Biophys Res Commun. (2007) 361:237–42. 10.1016/j.bbrc.2007.07.023 17658471

[38] Rana K Lee NK Zajac JD MacLean HE. Expression of androgen receptor target genes in skeletal muscle. Asian J Androl. (2014) 16:675–83. 10.4103/1008-682X.122861 24713826 PMC4215656

[39] Pronsato L Milanesi L Vasconsuelo A. Testosterone induces up-regulation of mitochondrial gene expression in murine C2C12 skeletal muscle cells accompanied by an increase of nuclear respiratory factor-1 and its downstream effectors. Mol Cell Endocrinol. (2020) 500:110631. 10.1016/j.mce.2019.110631 31676390

[40] Kwak JY Kwon KS. Pharmacological interventions for treatment of sarcopenia: current status of drug development for sarcopenia. Ann Geriatr Med Res. (2019) 23:98–104. 10.4235/agmr.19.0028 32743297 PMC7370765

[41] Dennison EM Sayer AA Cooper C. Epidemiology of sarcopenia and insight into possible therapeutic targets. Nat Rev Rheumatol. (2017) 13:340–7. 10.1038/nrrheum.2017.60 28469267 PMC5444517

[42] Burton LA Sumukadas D. Optimal management of sarcopenia. Clin Interv Aging. (2010) 5:217–28. 10.2147/cia.s11473 20852669 PMC2938029

[43] Serra C Tangherlini F Rudy S Lee D Toraldo G Sandor NL. Testosterone improves the regeneration of old and young mouse skeletal muscle. J Gerontol A Biol Sci Med Sci. (2013) 68:17–26. 10.1093/gerona/gls083 22499765 PMC3598367

[44] Ferrando AA Sheffield-Moore M Yeckel CW Gilkison C Jiang J Achacosa A. Testosterone administration to older men improves muscle function: molecular and physiological mechanisms. Am J Physiol Endocrinol Metab. (2002) 282:E601–7. 10.1152/ajpendo.00362.2001 11832363

[45] Martín AI Priego T López-Calderón A. Hormones and Muscle Atrophy. Adv Exp Med Biol. (2018) 1088:207–33. 10.1007/978-981-13-1435-3_9 30390253

[46] Morley JE Kaiser FE Perry HM. 3rd Patrick P Morley PM Stauber PM. Longitudinal changes in testosterone, luteinizing hormone, and follicle-stimulating hormone in healthy older men. Metabolism. (1997) 46:410–3. 10.1016/s0026-0495(97)90057-3 9109845

[47] Wang C Nieschlag E Swerdloff R Behre HM Hellstrom WJ Gooren LJ. Investigation, treatment and monitoring of late-onset hypogonadism in males. Int J Androl. (2009) 32:1–10. 10.1111/j.1365-2605.2008.00924.x 18798761

[48] Carrero JJ Qureshi AR Nakashima A Arver S Parini P Lindholm B. Prevalence and clinical implications of testosterone deficiency in men with end-stage renal disease. Nephrol Dial Transplant. (2011) 26:184–90. 10.1093/ndt/gfq397 20624775

[49] Sinclair M Grossmann M Gow PJ Angus PW. Testosterone in men with advanced liver disease: abnormalities and implications. J Gastroenterol Hepatol. (2015) 30:244–51. 10.1111/jgh.12695 25087838

[50] Kenny AM Kleppinger A Annis K Rathier M Browner B Judge JO. Effects of transdermal testosterone on bone and muscle in older men with low bioavailable testosterone levels, low bone mass, and physical frailty. J Am Geriatr Soc. (2010) 58:1134–43. 10.1111/j.1532-5415.2010.02865.x 20722847 PMC3014265

[51] Storer TW Basaria S Traustadottir T Harman SM Pencina K Li Z. Effects of testosterone supplementation for 3 years on muscle performance and physical function in older men. J Clin Endocrinol Metab. (2017) 102:583–93. 10.1210/jc.2016-2771 27754805 PMC5413164

[52] Wittert GA Chapman IM Haren MT Mackintosh S Coates P Morley JE. Oral testosterone supplementation increases muscle and decreases fat mass in healthy elderly males with low-normal gonadal status. J Gerontol A Biol Sci Med Sci. (2003) 58:618–25. 10.1093/gerona/58.7.m618 12865477

[53] Bauer JM Kaiser MJ Sieber CC. Sarcopenia in nursing home residents. J Am Med Dir Assoc. (2008) 9:545–51. 10.1016/j.jamda.2008.04.010 19083287

[54] Hohmann E Tetsworth K Hohmann S Bryant AL. Anabolic steroids after total knee arthroplasty. A double blinded prospective pilot study. J Orthop Surg Res. (2010) 5:93. 10.1186/1749-799X-5-93 21159157 PMC3009960

[55] Gharahdaghi N Rudrappa S Brook MS Idris I Crossland H Hamrock C. Testosterone therapy induces molecular programming augmenting physiological adaptations to resistance exercise in older men. J Cachexia Sarcopenia Muscle. (2019) 10:1276–94. 10.1002/jcsm.12472 31568675 PMC6903447

[56] Nam YS Lee G Yun JM Cho B. Testosterone replacement, muscle strength, and physical function. World J Mens Health. (2018) 36:110–22. 10.5534/wjmh.182001 29623702 PMC5924952

[57] Castro-Coronado J Yasima-Vásquez G Zapata-Lamana R Toloza-Ramírez D Cigarroa I. Characteristics of resistance training-based programs in older adults with sarcopenia: Scoping review. Rev Esp Geriatr Gerontol. (2021) 56:279–88. 10.1016/j.regg.2021.05.004 34147282

[58] Talar K Hernández-Belmonte A Vetrovsky T Steffl M Kałamacka E Courel-Ibáñez J. Benefits of resistance training in early and late stages of frailty and sarcopenia: a systematic review and meta-analysis of randomized controlled studies. J Clin Med. (2021) 10:1630. 10.3390/jcm10081630 33921356 PMC8070531

[59] Kvorning T Christensen LL Madsen K Nielsen JL Gejl KD Brixen K. Mechanical muscle function and lean body mass during supervised strength training and testosterone therapy in aging men with low-normal testosterone levels. J Am Geriatr Soc. (2013) 61:957–62. 10.1111/jgs.12279 23730808

[60] Sullivan DH Roberson PK Johnson LE Bishara O Evans WJ Smith ES. Effects of muscle strength training and testosterone in frail elderly males. Med Sci Sports Exerc. (2005) 37:1664–72. 10.1249/01.mss.0000181840.54860.8b 16260965

[61] Hildreth KL Barry DW Moreau KL Vande Griend J Meacham RB Nakamura T. Effects of testosterone and progressive resistance exercise in healthy, highly functioning older men with low-normal testosterone levels. J Clin Endocrinol Metab. (2013) 98:1891–900. 10.1210/jc.2013-2227 23533227 PMC3644594

[62] Falqueto H Júnior JLR Silvério MNO Farias JCH Schoenfeld BJ Manfredi LH. Can conditions of skeletal muscle loss be improved by combining exercise with anabolic-androgenic steroids? A systematic review and meta-analysis of testosterone-based interventions. Rev Endocr Metab Disord. (2021) 22:161–78. 10.1007/s11154-021-09634-4 33783694

[63] Holman ME Gorgey AS. Testosterone and resistance training improve muscle quality in spinal cord injury. Med Sci Sports Exerc. (2019) 51:1591–98. 10.1249/MSS.0000000000001975 30845047

[64] Wright TJ Dillon EL Durham WJ Chamberlain A Randolph KM Danesi C. A randomized trial of adjunct testosterone for cancer-related muscle loss in men and women. J Cachexia Sarcopenia Muscle. (2018) 9:482–96. 10.1002/jcsm.12295 29654645 PMC5989774

[65] Horwath O Apró W Moberg M Godhe M Helge T Ekblom M. Fiber type-specific hypertrophy and increased capillarization in skeletal muscle following testosterone administration in young women. J Appl Physiol. (2020) 128:1240–50. 10.1152/japplphysiol.00893.2019 32191598

[66] Tapper J Huang G Pencina KM Li Z Arver S Martling A. The effects of testosterone administration on muscle areas of the trunk and pelvic floor in hysterectomized women with low testosterone levels: proof-of-concept study. Menopause. (2019) 26:1405–14. 10.1097/GME.0000000000001410 31479032 PMC6893124

[67] Grech A Breck J Heidelbaugh J. Adverse effects of testosterone replacement therapy: an update on the evidence and controversy. Ther Adv Drug Saf. (2014) 5:190–200. 10.1177/2042098614548680 25360240 PMC4212439

[68] Budoff MJ Ellenberg SS Lewis CE Mohler ER 3rd Wenger NK Bhasin S. Testosterone treatment and coronary artery plaque volume in older men with low testosterone. JAMA. (2017) 317:708–16. 10.1001/jama.2016.21043 28241355 PMC5465430

[69] Grinspoon S Corcoran C Parlman K Costello M Rosenthal D Anderson E. Effects of testosterone and progressive resistance training in eugonadal men with AIDS wasting. A randomized, controlled trial. Ann Intern Med. (2000) 133:348–55. 10.7326/0003-4819-133-5-200009050-00010 10979879

[70] Fonseca GWPD Dworatzek E Ebner N Von Haehling S. Selective androgen receptor modulators (SARMs) as pharmacological treatment for muscle wasting in ongoing clinical trials. Expert Opin Investig Drugs. (2020) 29:881–91. 10.1080/13543784.2020.1777275 32476495

[71] Ostrowski J Kuhns JE Lupisella JA Manfredi MC Beehler BC Krystek SR Jr. Pharmacological and x-ray structural characterization of a novel selective androgen receptor modulator: potent hyperanabolic stimulation of skeletal muscle with hypostimulation of prostate in rats. Endocrinology. (2007) 148:4–12. 10.1210/en.2006-0843 17008401

[72] Komrakova M Furtwängler J Hoffmann DB Lehmann W Schilling AF Sehmisch S. The selective androgen receptor modulator ostarine improves bone healing in ovariectomized rats. Calcif Tissue Int. (2020) 106:147–57. 10.1007/s00223-019-00613-1 31531719

[73] Neil D Clark RV Magee M Billiard J Chan A Xue Z. GSK2881078, a SARM, produces dose-dependent increases in lean mass in healthy older men and women. J Clin Endocrinol Metab. (2018) 103:3215–24. 10.1210/jc.2017-02644 29982690

[74] Cozzoli A Capogrosso RF Sblendorio VT Dinardo MM Jagerschmidt C Namour F. GLPG0492, a novel selective androgen receptor modulator, improves muscle performance in the exercised-mdx mouse model of muscular dystrophy. Pharmacol Res. (2013) 72:9–24. 10.1016/j.phrs.2013.03.003 23523664

[75] Miner JN Chang W Chapman MS Finn PD Hong MH López FJ. An orally active selective androgen receptor modulator is efficacious on bone, muscle, and sex function with reduced impact on prostate. Endocrinology. (2007) 148:363–73. 10.1210/en.2006-0793 17023534

[76] Clark RV Walker AC Andrews S Turnbull P Wald JA Magee MH. Safety, pharmacokinetics and pharmacological effects of the selective androgen receptor modulator, GSK2881078, in healthy men and postmenopausal women. Br J Clin Pharmacol. (2017) 83:2179–94. 10.1111/bcp.13316 28449232 PMC5595940

[77] Basaria S Collins L Dillon EL Orwoll K Storer TW Miciek R. The safety, pharmacokinetics, and effects of LGD-4033, a novel nonsteroidal oral, selective androgen receptor modulator, in healthy young men. J Gerontol A Biol Sci Med Sci. (2013) 68:87–95. 10.1093/gerona/gls078 22459616 PMC4111291

[78] PapanicolaouDAAtherSNZhuHZhouYLutkiewiczJScottBB. A phase IIA randomized, placebo-controlled clinical trial to study the efficacy and safety of the selective androgen receptor modulator (SARM), MK-0773 in female participants with sarcopenia. J Nutr Health Aging. (2013) 17:533–43. 10.1007/s12603-013-0335-x23732550

[79] Dobs AS Boccia RV Croot CC Gabrail NY Dalton JT Hancock ML. Effects of enobosarm on muscle wasting and physical function in patients with cancer: a double-blind, randomised controlled phase 2 trial. Lancet Oncol. (2013) 14:335–45. 10.1016/S1470-2045(13)70055-X 23499390 PMC4898053

[80] Dalton JT Barnette KG Bohl CE Hancock ML Rodriguez D Dodson ST. The selective androgen receptor modulator GTx-024 (enobosarm) improves lean body mass and physical function in healthy elderly men and postmenopausal women: results of a double-blind, placebo-controlled phase II trial. J Cachexia Sarcopenia Muscle. (2011) 2:153–61. 10.1007/s13539-011-0034-6 22031847 PMC3177038

[81] Crawford J Prado CM Johnston MA Gralla RJ Taylor RP Hancock ML. Study design and rationale for the phase 3 clinical development program of enobosarm, a selective androgen receptor modulator, for the prevention and treatment of muscle wasting in cancer patients (POWER Trials). Curr Oncol Rep. (2016) 18:37. 10.1007/s11912-016-0522-0 27138015 PMC4853438

[82] Ramage MI Skipworth RJE. The relationship between muscle mass and function in cancer cachexia: smoke and mirrors? Curr Opin Support Palliat Care. (2018) 12:439–44. 10.1097/SPC.0000000000000381 30138131

[83] Ikeda K Horie-Inoue K Inoue S. Functions of estrogen and estrogen receptor signaling on skeletal muscle. J Steroid Biochem Mol Biol. (2019) 191:105375. 10.1016/j.jsbmb.2019.105375 31067490

[84] Pöllänen E Sipilä S Alen M Ronkainen PH Ankarberg-Lindgren C Puolakka J. Differential influence of peripheral and systemic sex steroids on skeletal muscle mass in pre- and postmenopausal women. Aging Cell. (2011) 10:650–60. 10.1111/j.1474-9726.2011.00701.x 21388496

[85] Zhang Y Xu Z Zhang J Tang J Liu F Song Y. 17-β-estradiol and progesterone as efficient predictors of survival in older women undergoing hip fracture surgery. Front Med. (2020) 7:345. 10.3389/fmed.2020.00345 32850880 PMC7426436

[86] Dumont NA Bentzinger CF Sincennes MC Rudnicki MA. Satellite cells and skeletal muscle regeneration. Compr Physiol. (2015) 5:1027–59. 10.1002/cphy.c140068 26140708

[87] Anderson LJ Liu H Garcia JM. Sex differences in muscle wasting. Adv Exp Med Biol. (2017) 1043:153–97. 10.1007/978-3-319-70178-3_9 29224095

[88] Sakuma K Yamaguchi A. Sarcopenia and age-related endocrine function. Int J Endocrinol. (2012) 2012:127362. 10.1155/2012/127362 22690213 PMC3368374

[89] Kenny AM Kleppinger A Wang Y Prestwood KM. Effects of ultra-low-dose estrogen therapy on muscle and physical function in older women. J Am Geriatr Soc. (2005) 53:1973–7. 10.1111/j.1532-5415.2005.53567.x 16274381

[90] La Colla A Pronsato L Milanesi L Vasconsuelo A. 17β-Estradiol and testosterone in sarcopenia: Role of satellite cells. Ageing Res Rev. (2015) 24:166–77. 10.1016/j.arr.2015.07.011 26247846

[91] Haizlip KM Harrison BC Leinwand LA. Sex-based differences in skeletal muscle kinetics and fiber-type composition. Physiology. (2015) 30:30–9. 10.1152/physiol.00024.2014 25559153 PMC4285578

[92] Moran AL Nelson SA Landisch RM Warren GL Lowe DA. Estradiol replacement reverses ovariectomy-induced muscle contractile and myosin dysfunction in mature female mice. J Appl Physiol. (2007) 102:1387–93. 10.1152/japplphysiol.01305.2006 17218423

[93] Sipilä S Narici M Kjaer M Pöllänen E Atkinson RA Hansen M. Sex hormones and skeletal muscle weakness. Biogerontology. (2013) 14:231–45. 10.1007/s10522-013-9425-8 23636830

[94] Greising SM Baltgalvis KA Lowe DA Warren GL. Hormone therapy and skeletal muscle strength: a meta-analysis. J Gerontol A Biol Sci Med Sci. (2009) 64:1071–81. 10.1093/gerona/glp082 19561145 PMC2737591

[95] Sørensen MB Rosenfalck AM Højgaard L Ottesen B. Obesity and sarcopenia after menopause are reversed by sex hormone replacement therapy. Obes Res. (2001) 9:622–6. 10.1038/oby.2001.81 11595778

[96] Taaffe DR Newman AB Haggerty CL Colbert LH de Rekeneire N Visser M. Estrogen replacement, muscle composition, and physical function: the health ABC study. Med Sci Sports Exerc. (2005) 37:1741–7. 10.1249/01.mss.0000181678.28092.31 16260975

[97] Tiidus PM. Benefits of estrogen replacement for skeletal muscle mass and function in post-menopausal females: evidence from human and animal studies. Eurasian J Med. (2011) 43:109–14. 10.5152/eajm.2011.24 25610174 PMC4261347

[98] Pöllänen E Ronkainen PH Suominen H Takala T Koskinen S Puolakka J. Muscular transcriptome in postmenopausal women with or without hormone replacement. Rejuvenation Res. (2007) 10:485–500. 10.1089/rej.2007.0536 17985945

[99] Sipilä S Taaffe DR Cheng S Puolakka J Toivanen J Suominen H. Effects of hormone replacement therapy and high-impact physical exercise on skeletal muscle in post-menopausal women: a randomized placebo-controlled study. Clin Sci. (2001) 101:147–57. 10.1042/cs1010147 11473488

[100] Thorneycroft IH Lindsay R Pickar JH. Body composition during treatment with conjugated estrogens with and without medroxyprogesterone acetate: analysis of the Women's Health, Osteoporosis, Progestin, Estrogen (HOPE) trial. Am J Obstet Gynecol. (2007) 197:137.e1–e7. 10.1016/j.ajog.2007.05.042 17689624

[101] Javed AA Mayhew AJ Shea AK Raina P. Association between hormone therapy and muscle mass in postmenopausal women: a systematic review and meta-analysis. JAMA Netw Open. (2019) 2:e1910154. 10.1001/jamanetworkopen.2019.10154 31461147 PMC6716293

[102] vandenBerg P Neumark-Sztainer D Cafri G Wall M. Steroid use among adolescents: longitudinal findings from Project EAT. Pediatrics. (2007) 119:476–86. 10.1542/peds.2006-2529 17332200

[103] Ettinger B Quesenberry C Schroeder DA Friedman G. Long-term postmenopausal estrogen therapy may be associated with increased risk of breast cancer: a cohort study. Menopause. (2018) 25:1191–94. 10.1097/GME.0000000000001216 30358712

[104] Chlebowski RT Hendrix SL Langer RD Stefanick ML Gass M Lane D. Influence of estrogen plus progestin on breast cancer and mammography in healthy postmenopausal women: the Women's Health Initiative Randomized Trial. JAMA. (2003) 289:3243–53. 10.1001/jama.289.24.3243 12824205

[105] Khovidhunkit W Shoback DM. Clinical effects of raloxifene hydrochloride in women. Ann Intern Med. (1999) 130:431–9. 10.7326/0003-4819-130-5-199903020-00015 10068418

[106] Ott SM Oleksik A Lu Y Harper K Lips P. Bone histomorphometric and biochemical marker results of a 2-year placebo-controlled trial of raloxifene in postmenopausal women. J Bone Miner Res. (2002) 17:341–8. 10.1359/jbmr.2002.17.2.341 11811565

[107] Dieli-Conwright CM Spektor TM Rice JC Todd Schroeder E. Oestradiol and SERM treatments influence oestrogen receptor coregulator gene expression in human skeletal muscle cells. Acta Physiol. (2009) 197:187–96. 10.1111/j.1748-1716.2009.01997.x 19432593

[108] Meli R Pacilio M Raso GM Esposito E Coppola A Nasti A. Estrogen and raloxifene modulate leptin and its receptor in hypothalamus and adipose tissue from ovariectomized rats. Endocrinology. (2004) 145:3115–21. 10.1210/en.2004-0129 15059958

[109] Jacobsen DE Samson MM Emmelot-Vonk MH Verhaar HJ. Raloxifene and body composition and muscle strength in postmenopausal women: a randomized, double-blind, placebo-controlled trial. Eur J Endocrinol. (2010) 162:371–6. 10.1530/EJE-09-0619 19884264

[110] Francucci CM Daniele P Iori N Camilletti A Massi F Boscaro M. Effects of raloxifene on body fat distribution and lipid profile in healthy post-menopausal women. J Endocrinol Invest. (2005) 28:623–31. 10.1007/BF03347261 16218045

[111] Urano T Shiraki M Kuroda T Tanaka S Uenishi K Inoue S. Preventive effects of raloxifene treatment on agerelated weight loss in postmenopausal women. J Bone Miner Metab. (2017) 35:108–13. 10.1007/s00774-015-0733-8 26754796

[112] Maggio M Lauretani F Ceda GP. Sex hormones and sarcopenia in older persons. Curr Opin Clin Nutr Metab Care. (2013) 16:3–13. 10.1097/MCO.0b013e32835b6044 23222704

[113] Huang K Cai HL Bao JP Wu LD. Dehydroepiandrosterone and age-related musculoskeletal diseases: Connections and therapeutic implications. Ageing Res Rev. (2020) 62:101132. 10.1016/j.arr.2020.101132 32711158

[114] Corona G Rastrelli G Giagulli VA Sila A Sforza A Forti G. Dehydroepiandrosterone supplementation in elderly men: a meta-analysis study of placebo-controlled trials. J Clin Endocrinol Metab. (2013) 98:3615–26. 10.1210/jc.2013-1358 23824417

[115] Ceci R Duranti G Rossi A Savini I Sabatini S. Skeletal muscle differentiation: role of dehydroepiandrosterone sulfate. Horm Metab Res. (2011) 43:702–7. 10.1055/s-0031-1285867 21932174

[116] Vitale G Cesari M Mari D. Aging of the endocrine system and its potential impact on sarcopenia. Eur J Intern Med. (2016) 35:10–5. 10.1016/j.ejim.2016.07.017 27484963

[117] Consitt LA Copeland JL Tremblay MS. Endogenous anabolic hormone responses to endurance versus resistance exercise and training in women. Sports Med. (2002) 32:1–22. 10.2165/00007256-200232010-00001 11772159

[118] Maninger N Wolkowitz OM Reus VI Epel ES Mellon SH. Neurobiological and neuropsychiatric effects of dehydroepiandrosterone (DHEA) and DHEA sulfate (DHEAS). Front Neuroendocrinol. (2009) 30:65–91. 10.1016/j.yfrne.2008.11.002 19063914 PMC2725024

[119] van den Beld AW Kaufman JM Zillikens MC Lamberts SWJ Egan JM. The physiology of endocrine systems with ageing. Lancet Diabetes Endocrinol. (2018) 6:647–58. 10.1016/S2213-8587(18)30026-3 30017799 PMC6089223

[120] Valenti G Denti L Maggio M Ceda G Volpato S Bandinelli S. Effect of DHEAS on skeletal muscle over the life span: the InCHIANTI study. J Gerontol A Biol Sci Med Sci. (2004) 59:466–72. 10.1093/gerona/59.5.m466 15123757

[121] Kostka T Arsac LM Patricot MC Berthouze SE Lacour JR Bonnefoy M. Leg extensor power and dehydroepiandrosterone sulfate, insulin-like growth factor-I and testosterone in healthy active elderly people. Eur J Appl Physiol. (2000) 82:83–90. 10.1007/s004210050655 10879447

[122] Morales AJ Haubrich RH Hwang JY Asakura H Yen SS. The effect of six months treatment with a 100 mg daily dose of dehydroepiandrosterone (DHEA) on circulating sex steroids, body composition and muscle strength in age-advanced men and women. Clin Endocrinol. (1998) 49:421–32. 10.1046/j.1365-2265.1998.00507.x 9876338

[123] Villareal DT Holloszy JO Kohrt WM. Effects of DHEA replacement on bone mineral density and body composition in elderly women and men. Clin Endocrinol. (2000) 53:561–8. 10.1046/j.1365-2265.2000.01131.x 11106916

[124] Villareal DT Holloszy JO. Effect of DHEA on abdominal fat and insulin action in elderly women and men: a randomized controlled trial. JAMA. (2004) 292:2243–8. 10.1001/jama.292.18.2243 15536111

[125] Percheron G Hogrel JY Denot-Ledunois S Fayet G Forette F Baulieu EE. Double-blind placebo-controlled trial. Effect of 1-year oral administration of dehydroepiandrosterone to 60- to 80-year-old individuals on muscle function and cross-sectional area: a double-blind placebo-controlled trial. Arch Intern Med. (2003) 163:720–7. 10.1001/archinte.163.6.720 12639206

[126] Igwebuike A Irving BA Bigelow ML Short KR McConnell JP Nair KS. Lack of dehydroepiandrosterone effect on a combined endurance and resistance exercise program in postmenopausal women. J Clin Endocrinol Metab. (2008) 93:534–8. 10.1210/jc.2007-1027 18029465 PMC2729150

[127] Baker WL Karan S Kenny AM. Effect of dehydroepiandrosterone on muscle strength and physical function in older adults: a systematic review. J Am Geriatr Soc. (2011) 59:997–1002. 10.1111/j.1532-5415.2011.03410.x 21649617

[128] BeaudartCRabendaVSimmonsMGeerinckAAraujo De CarvalhoIReginsterJY. Effects of protein, essential amino acids, b-hydroxy b-methylbutyrate, creatine, dehydroepiandrosterone and fatty acid supplementation on muscle mass, muscle strength and physical performance in older people aged 60 years and over. A systematic review on the literature. J Nutr Health Aging. (2018) 22:117–30. 10.1007/s12603-017-0934-z29300431

[129] Sato K Iemitsu M Aizawa K Mesaki N Ajisaka R Fujita S. administration and exercise training improves insulin resistance in obese rats. Nutr Metab. (2012) 9:47. 10.1186/1743-7075-9-47 22647230 PMC3433349

[130] Villareal DT Holloszy JO DHEA. enhances effects of weight training on muscle mass and strength in elderly women and men. Am J Physiol Endocrinol Metab. (2006) 291:E1003–8. 10.1152/ajpendo.00100.2006 16787962

[131] Traish AM Kang HP Saad F Guay AT. Dehydroepiandrosterone (DHEA)–a precursor steroid or an active hormone in human physiology. J Sex Med. (2011) 8:2960–82. 10.1111/j.1743-6109.2011.02523.x 22032408

[132] Labrie F. DHEA important source of sex steroids in men and even more in women. Prog Brain Res. (2010) 182:97–148. 10.1016/S0079-6123(10)82004-7 20541662

[133] Taraborrelli S. Physiology, production and action of progesterone. Acta Obstet Gynecol Scand. (2015) 161:8–16. 10.1111/aogs.12771 26358238

[134] Ekenros L Papoutsi Z Fridén C Dahlman Wright K Lindén Hirschberg A. Expression of sex steroid hormone receptors in human skeletal muscle during the menstrual cycle. Acta Physiol. (2017) 219:486–93. 10.1111/apha.12757 27438889

[135] Wiik A Ekman M Johansson O Jansson E Esbjörnsson M. Expression of both oestrogen receptor alpha and beta in human skeletal muscle tissue. Histochem Cell Biol. (2009) 131:181–9. 10.1007/s00418-008-0512-x 18825402

[136] Hansen M. Female hormones: do they influence muscle and tendon protein metabolism? Proc Nutr Soc. (2018) 77:32–41. 10.1017/S0029665117001951 28847313

[137] Yeh SS Wu SY Lee TP Olson JS Stevens MR Dixon T. Improvement in quality-of-life measures and stimulation of weight gain after treatment with megestrol acetate oral suspension in geriatric cachexia: results of a double-blind, placebo-controlled study. J Am Geriatr Soc. (2000) 48:485–92. 10.1111/j.1532-5415.2000.tb04993.x 10811540

[138] Tchekmedyian NS Hickman M Siau J Greco FA Keller J Browder H. Megestrol acetate in cancer anorexia and weight loss. Cancer. (1992) 69:1268–74. 10.1002/cncr.2820690532 1739926

[139] Sullivan DH Roberson PK Smith ES Price JA Bopp MM. Effects of muscle strength training and megestrol acetate on strength, muscle mass, and function in frail older people. J Am Geriatr Soc. (2007) 55:20–8. 10.1111/j.1532-5415.2006.01010.x 17233681

[140] Taaffe DR Sipilä S Cheng S Puolakka J Toivanen J Suominen H. The effect of hormone replacement therapy and/or exercise on skeletal muscle attenuation in postmenopausal women: a yearlong intervention. Clin Physiol Funct Imaging. (2005) 25:297–304. 10.1111/j.1475-097X.2005.00628.x 16117734

[141] Romance R Vargas S Espinar S Petro JL Bonilla DA Schöenfeld BJ. Oral contraceptive use does not negatively affect body composition and strength adaptations in trained women. Int J Sports Med. (2019) 40:842–49. 10.1055/a-0985-4373 31491790

[142] Riechman SE Lee CW. Oral contraceptive use impairs muscle gains in young women. J Strength Cond Res. (2021). 10.1519/JSC.0000000000004059. [Epub ahead of print]. 33993156

[143] Fournier A Berrino F Clavel-Chapelon F. Unequal risks for breast cancer associated with different hormone replacement therapies: results from the E3N cohort study. Breast Cancer Res Treat. (2008) 107:103–11. 10.1007/s10549-007-9523-x 17333341 PMC2211383

